# Proteomic and Transcriptomic Analysis of *Microviridae* φX174 Infection Reveals Broad Upregulation of Host Escherichia coli Membrane Damage and Heat Shock Responses

**DOI:** 10.1128/mSystems.00046-21

**Published:** 2021-05-11

**Authors:** Bradley W. Wright, Dominic Y. Logel, Mehdi Mirzai, Dana Pascovici, Mark P. Molloy, Paul R. Jaschke

**Affiliations:** a Department of Molecular Sciences, Macquarie University, Sydney, NSW, Australia; b Australian Proteome Analysis Facility (APAF), Macquarie University, Sydney, NSW, Australia; c Kolling Institute, Northern Clinical School, The University of Sydney, Sydney, NSW, Australia; Vanderbilt University

**Keywords:** bacteriophage, chaperone, gut microbiome, lipoprotein, mass spectrometry, RNA-seq, small heat shock proteins, sHSP, chaperones, microbiome

## Abstract

Measuring host-bacteriophage dynamics is an important approach to understanding bacterial survival functions and responses to infection. The model *Microviridae* bacteriophage φX174 is endemic to the human gut and has been studied for over 70 years, but the host response to infection has never been investigated in detail. To address this gap in our understanding of this important interaction within our microbiome, we have measured host Escherichia coli C proteomic and transcriptomic response to φX174 infection. We used mass spectrometry and RNA sequencing (RNA-seq) to identify and quantify all 11 φX174 proteins and over 1,700 E. coli proteins, enabling us to comprehensively map host pathways involved in φX174 infection. Most notably, we see significant host responses centered on membrane damage and remodeling, cellular chaperone and translocon activity, and lipoprotein processing, which we speculate is due to the peptidoglycan-disruptive effects of the φX174 lysis protein E on MraY activity. We also observe the massive upregulation of small heat shock proteins IbpA/B, along with other heat shock pathway chaperones, and speculate on how the specific characteristics of holdase protein activity may be beneficial for viral infections. Together, this study enables us to begin to understand the proteomic and transcriptomic host responses of E. coli to *Microviridae* infections and contributes insights to the activities of this important model host-phage interaction.

**IMPORTANCE** A major part of the healthy human gut microbiome is the *Microviridae* bacteriophage, exemplified by the model φX174 phage, and their E. coli hosts. Although much has been learned from studying φX174 over the last half-century, until this work, the E. coli host response to infection has never been investigated in detail. We reveal the proteomic and transcriptomic pathways differentially regulated during the φX174 infection cycle and uncover the details of a coordinated cellular response to membrane damage that results in increased lipoprotein processing and membrane trafficking, likely due to the phage antibiotic-like lysis protein. We also reveal that small heat shock proteins IbpA/B are massively upregulated during infection and that these holdase chaperones are highly conserved across the domains of life, indicating that reliance on them is likely widespread across viruses.

## Abstract (precis)

A major part of the healthy human gut microbiome is the *Microviridae* bacteriophage, exemplified by the model φX174 phage, and their E. coli hosts. Although much has been learned from studying φX174 over the last half-century, until this work, the E. coli host response to infection has never been investigated in detail.

## INTRODUCTION

Successful phage reproduction relies on suppression and evasion of host defense responses (1) and, to various extents, requisitioning host machinery (2). Temporal infection studies employing the use of transcriptomics (3, 4) and proteomics (5–11) have revealed insights into the establishment of infection and utilization of host machinery. In particular, the use of modern mass spectrometry workflows and instruments to identify and quantify thousands of proteins is an invaluable tool for the study of complex biological systems especially when paired with complementary RNA-sequencing (RNA-seq) methods. Currently, most phage-host interactions, even of model systems, have not yet been characterized using these methods.

Bacteriophage φX174 is a member of the family *Microviridae*, which make up a group of small icosahedral viruses encoded by a single-stranded DNA genome. The small 5,386-nucleotide genome of φX174 containing genes encoding 11 proteins makes it a tractable target for proteomic and transcriptomic studies (12, 13). *Microviridae* have been used as model systems for DNA sequencing, genome engineering (14, 15), evolution (16), icosahedral virus packaging (17, 18), and novel virus infection mechanisms (19, 20). Furthermore, φX174 and other *Microviridae* are universal members of the human gut microbiome (21, 22), preying on Escherichia coli hosts, although their precise role is not well understood.

In this investigation, we applied modern proteomics and complementary transcriptomics to map for the first time the temporal response of E. coli NCTC122 (C122) to phage φX174 infection. Our detailed measurements of protein and RNA revealed the heat shock chaperone network is selectively upregulated during infection, along with many proteins involved in membrane repair and remodeling and membrane transporters. Most notably, we observed massive upregulation of small heat shock proteins IbpA and IbpB at both the protein and RNA level. This work lays the foundation for further studies of *Microviridae* infections and their interplay within a range of more natural host strains.

## RESULTS

### φX174 infection results in moderate changes to the host proteome and transcriptome just prior to lysis.

To measure the host response to φX174 infection, we first used a mass spectrometry proteomic workflow to compare biological triplicates of φX174-infected E. coli C122 and mock-infected cells at five time points (0, 15, 30, 60, and 75 min postinfection) designed to maximize coverage across the entire period leading up to and just following the onset of lysis. We found no differences between the host proteome expression and mock infections until 60 and 75 min, corresponding to the point at which we observed reduction in the rate of *A*_600_ increase characteristic of the beginning of lysis (see Fig. S1A in the supplemental material). Mass spectrometry analysis identified 2,173 host proteins (Data Set S1), representing ∼57% of the E. coli C122 proteome database. Of the identified proteins, we measured the expression of 1,752 host proteins across all time points and replicates as well as all 11 φX174 proteins. For these proteins, due to the different physicochemical properties of the peptides and the acquisition strategy used (23), we were limited to relative quantitation expressed as fold change (FC), which was calculated by dividing normalized area under the curve intensity measurements from condition 1 by condition 2.

10.1128/mSystems.00046-21.1 FIG S1 Mock- and φX174-infected E. coli C122 analysis. (A) Lysis curve of φX174 and E. coli C122. Cell lysis caused by φX174 infection as inferred by the reduction of culture OD_600_. Each point is the average from three biological replicates with error bars representing 1 standard deviation. (B) Two-dimensional principal-component analysis (PCA) of mock-infected (C) and φX174-infected (P) samples. Separate clustering of the later time point control samples (Late mock) to that of the early time points of both mock-infected and φX174-infected (Early) can be observed. Similarly, separate clustering of later time points of the 60-min and 75-min φX174-infected samples (60P and 75P) to each other and of the late mock and early groupings is observed. (C) Hierarchical clustering with Euclidean distance of quantified proteins. Clustering of later time points (late) and their associated condition (mock = mock infected, inf. = φX174 infected) highlight the change in the proteome over time and by φX174 infection. Download FIG S1, EPS file, 1.6 MB. Copyright © 2021 Wright et al. 2021 Wright et al. https://creativecommons.org/licenses/by/4.0/ This content is distributed under the terms of the Creative Commons Attribution 4.0 International license.

10.1128/mSystems.00046-21.7 DATA SET S1 TMT proteomics data. Download Data Set S1, XLSX file, 1.6 MB. Copyright © 2021 Wright et al. 2021 Wright et al. https://creativecommons.org/licenses/by/4.0/ This content is distributed under the terms of the Creative Commons Attribution 4.0 International license.

To further validate our proteomics work, we also measured the transcriptional response of the host at 60 min. RNA sequencing mapped reads to 85% of E. coli protein-encoding genes and 100% of φX174 genes. Furthermore, during infection, phage-specific reads accounted for 18% of the total mapped reads.

**FIG 1:**
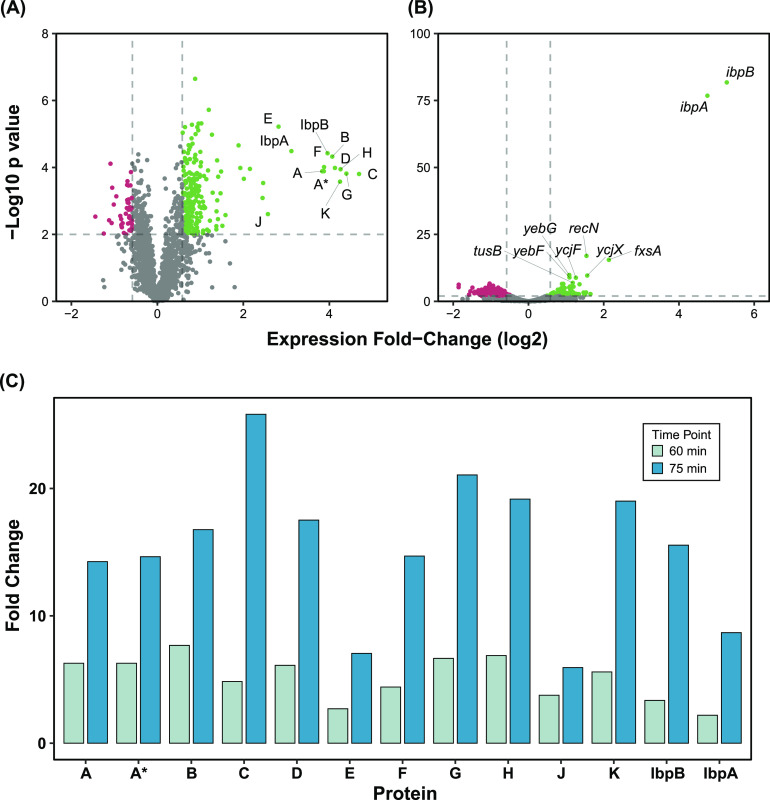
A moderate number of E. coli C122 genes are differentially regulated during late φX174 infection, with *ibpAB* upregulation equal to φX174 phage genes. (A) Quantified proteins. FC ±1.5 and *P* value <0.025 significance threshold. (B) Quantified RNA. FC ±1.5 and *P* value cutoff <0.05 significance threshold. (C) Fold changes for φX174 proteins labeled A to K and host E. coli proteins IbpA and IbpB at 60 min and 75 min postinfection. Fold change reflects the ratio of quantified values of infected to mock samples.

During late infection we measured at the protein level 255 upregulated and 54 downregulated proteins (Fig. 1A; Data Set S1), while at the RNA level 203 genes were found to be upregulated and 325 genes were downregulated (Fig. 1B; Fig. S2). The *ibpA* and *ibpB* small heat shock proteins, which had FCs of +8.7/+27.1 and +15.6/+38.6 at the protein/RNA levels, respectively, were similar in magnitude and significance to the φX174 proteins (Fig. 1C). Notably, we did not observe any significant changes to small RNAs within E. coli C122 due to φX174 infection (Data Set S2).

10.1128/mSystems.00046-21.2 FIG S2 Differential expression of E. coli C122 genes during φX174 infection. (A) Differential expression of all genes within infected E. coli C122 with significantly upregulated genes shown in green and significantly downregulated genes shown in red. Dashed lines represent significance criteria (log_2_ fold change = ±0.585 and *P* values < 0.05). (B) Differentially expressed genes excluding highly upregulated genes *ibpA* and *ibpB*. (C) Downregulated genes (closeup). (D) Upregulated genes (closeup). Comparisons were made between RNA-sequencing outputs from cultures harvested at lysis in the infected and mock-infected samples; samples were analyzed using Rsubread and DESeq2 in biological triplicates. Download FIG S2, EPS file, 1.1 MB. Copyright © 2021 Wright et al. 2021 Wright et al. https://creativecommons.org/licenses/by/4.0/ This content is distributed under the terms of the Creative Commons Attribution 4.0 International license.

10.1128/mSystems.00046-21.8 DATA SET S2 Transcriptional data set of all C122 genes. Download Data Set S2, XLSX file, 0.6 MB. Copyright © 2021 Wright et al. 2021 Wright et al. https://creativecommons.org/licenses/by/4.0/ This content is distributed under the terms of the Creative Commons Attribution 4.0 International license.

### Host engages in significant membrane protein upregulation and metabolic protein downregulation during infection.

To define the broader biological impacts of φX174 infection, we assigned all 1,752 quantified host proteins, 11 φX174 proteins, and 528 differentially expressed RNA transcripts to their respective clusters of orthologous groupings (COG) using eggNOGMapper (24).

**FIG 2:**
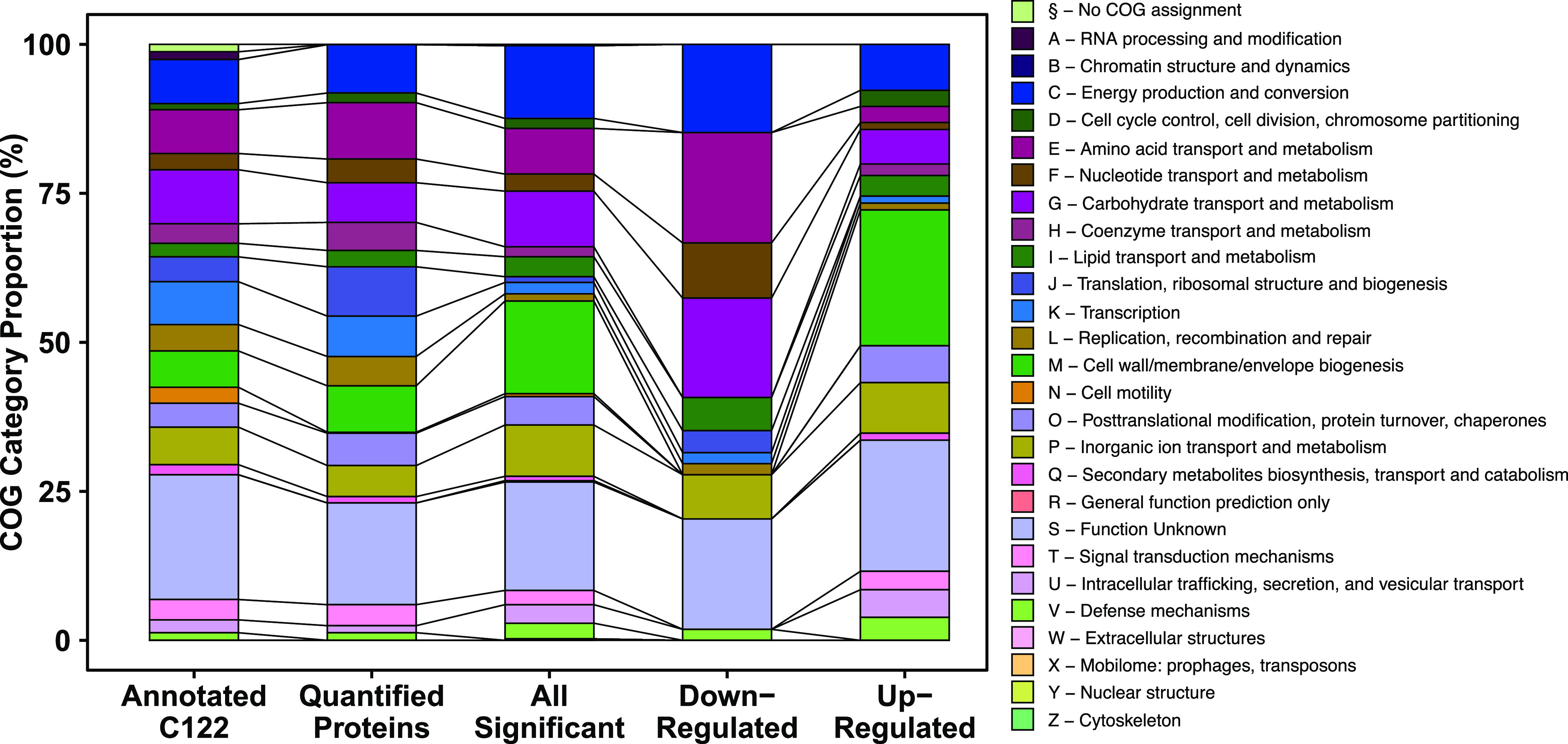
Clusters of orthologous groups (COG) distributions of measured E. coli C122 proteins. Annotated C122, 3,666 annotated proteins; Quantified Proteins, 1,752 proteins quantified across all conditions; All Significant, nonredundant list of 399 significantly differentially regulated proteins across all time points and samples; Down-Regulated, 54 significantly downregulated proteins from 75 min postinfection; Up-Regulated, 255 significantly upregulated proteins from 75 min postinfection. COGs were assigned to C122 genes using the eggNOG-Mapper (24).

The results of this analysis showed no proteomic sample preparation enrichment bias as the “Annotated C122” and “Quantified Proteins” annotated biological function category COG distributions were comparable (Fig. 2). We then partitioned the 309 significantly differentially regulated proteins at 75 min postinfection into those that were upregulated (“Up-Regulated,” *n* = 255) and those that were downregulated (“Down-Regulated,” *n* = 54). We found that all proteins annotated with COG categories M (cell wall/membrane/envelope biogenesis) and O (posttranslational modification, protein turnover, chaperones) were upregulated during infection (Fig. 2). These category enrichments indicate there may be a host response mediating significant membrane restructuring or poor translocation of membrane-bound proteins leading to their accumulation in the cytoplasm. Within the group of 54 downregulated proteins, we found the majority of these proteins (∼59%) were in COG categories representative of energy and metabolism (C, E, F, and G) (Fig. 2).

Using the PANTHER overrepresentation test (25), we mapped these proteins according to their GO cellular component annotations. We found that of the upregulated proteins, 167 (65%) could be mapped to a cellular component, of which 21 (13%) were localized to the cytoplasm. These included small heat shock proteins IbpA and IbpB. Similarly, 39 (72%) of the downregulated proteins could be mapped to their cellular component, and 24 (62%) of these were mapped to the cytoplasm.

Corresponding with the proteomic findings, the transcriptomics showed COG category enrichment for inorganic ion transport and metabolism (P), amino acid transport and metabolism (E), and energy production and conversion (C) (Fig. S3). Additionally, the transcriptomics showed enrichment for the COG categories cell motility (N) and intracellular trafficking, secretion, and vesicular transport (U). PANTHER overrepresentation testing for GO cellular component terms showed significant enrichment within upregulated genes for the pilus (6.7-fold) and the integral component of the membrane (1.6-fold). The downregulated genes were enriched for the iron-sulfur cluster assembly complex (16.2-fold), the proton-transporting ATP synthase complex, catalytic core F(1) (13.5-fold), and the plasma membrane respiratory chain complex I (11.2-fold) (Fig. S4). All of these enriched cellular components are localized within the bacterial membrane. Membrane component enrichment was reflected in the differentially expressed genes more broadly as well, as gene products destined for the membrane accounted for 42% and 24% of the up- and downregulated data sets, respectively.

10.1128/mSystems.00046-21.3 FIG S3 Biological function COG distributions for differentially expressed E. coli C122 genes in the RNA-seq data set. The enrichment of COG terms in the down- and upregulated RNA-seq data sets from C122 at lysis. UniProt IDs mapped to COG terms using eggNOG-Mapper. Gene IDs with multiple COG terms were converted to multiple single-term entries for analysis. Download FIG S3, EPS file, 1.8 MB. Copyright © 2021 Wright et al. 2021 Wright et al. https://creativecommons.org/licenses/by/4.0/ This content is distributed under the terms of the Creative Commons Attribution 4.0 International license.

10.1128/mSystems.00046-21.4 FIG S4 Major biological function GO terms differentially expressed in RNA-seq data set. Differentially expressed genes from E. coli C122 were analyzed using the PANTHER overrepresentation test using the GO terms for Biological Function. All presented data displayed a *P* value of <0.01. Bolded terms represent child functions with associated parent function(s) in the same color below. Download FIG S4, PDF file, 1.1 MB. Copyright © 2021 Wright et al. 2021 Wright et al. https://creativecommons.org/licenses/by/4.0/ This content is distributed under the terms of the Creative Commons Attribution 4.0 International license.

**FIG 3:**
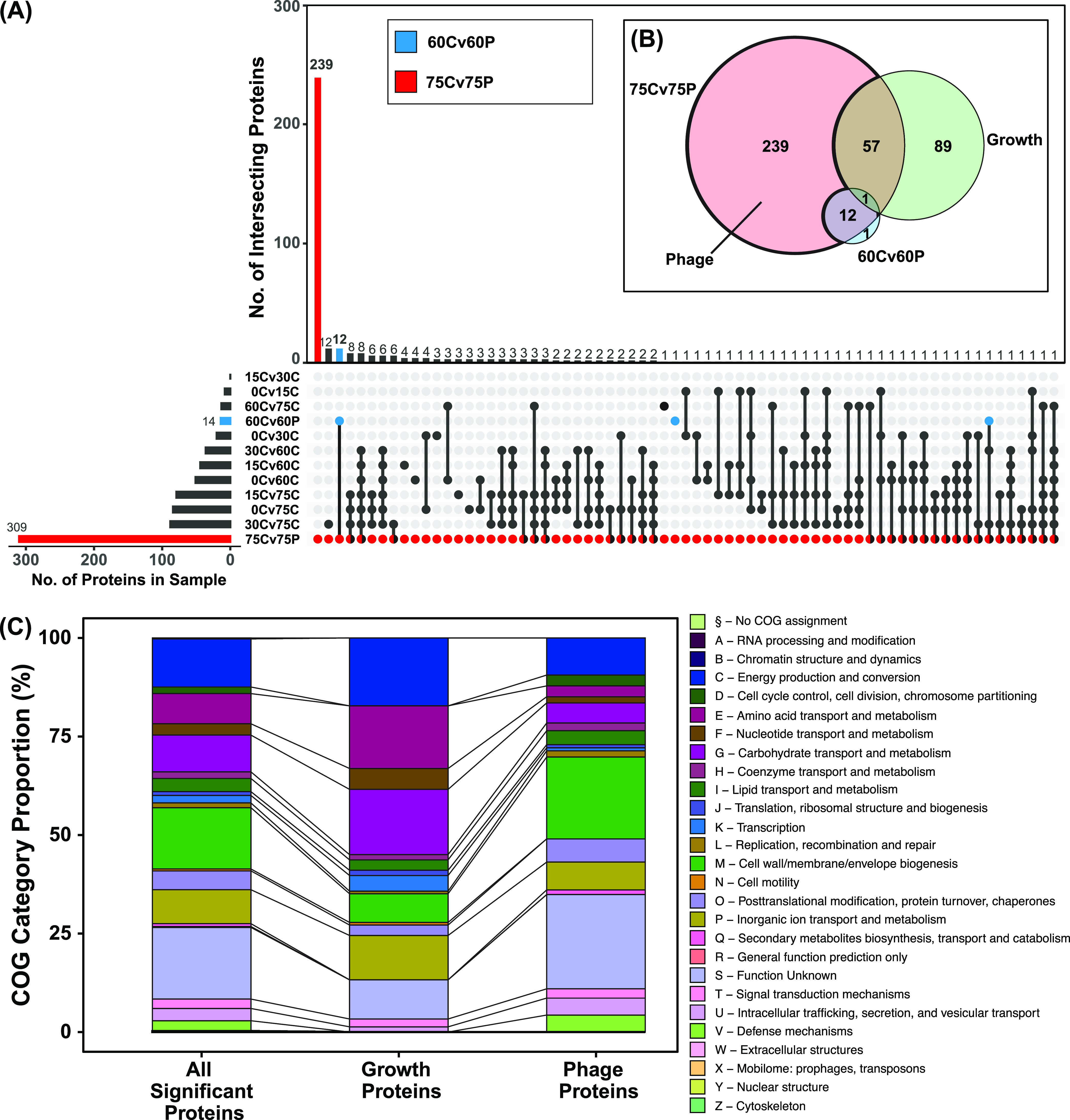
Sets of significantly differentially regulated proteins due to cellular growth and φX174 infection and their functional groupings. (A) UpSet visualization of sample groups analyzed by proteomics. Each set represents the group of significantly regulated proteins (FC ±1.5, *P* value <0.025) extracted from comparative analyses. For example, 75Cv75P (red) is the group of significant proteins passing our stated cutoff thresholds when the 75-min mock-infected (75C) group is compared to the 75-min φX174-infected (75P) group. This plot allows visualization of redundant proteins found differentially regulated across multiple groups and, more importantly, facilitates the partitioning out of significant proteins relevant only to φX174 infection. Nonredundant sample size = 399 proteins. (B) Euler plot showing relationships between Phage, Growth, 60Cv60P, and 75Cv75P protein sets. (C) Clusters of orthologous groups (COG) categories of the Growth and Phage protein sets compared to 399 significant proteins (“All Significant Proteins”). See Data Set S1. COGs were assigned to C122 genes using the eggNOG-Mapper (24).

To understand the interaction of φX174 protein expression and host responses to infection, we performed comparative analyses using an UpSet plot, which is a tool to visualize and group shared elements within intersecting data sets (26). We used the UpSet visualization to identify the conditions and samples in which significantly differentially regulated proteins are shared. This analysis revealed that the 60-min-postinfection samples (60C and 60P) shared 12 differentially expressed proteins with the 75-min-postinfection samples (75C and 75P): two host proteins (IbpA and IbpB), and all φX174 proteins except C protein (Fig. 3A; Data Set S1).

In contrast to the 60-min samples, the 75-min-postinfection samples showed large-scale changes to the host proteome, with 309 differentially regulated proteins including all 11 phage proteins and the same two host proteins, IbpA and IbpB, seen at 60 min (Fig. 3A). Within this group of 309 proteins, we wanted to identify proteins that were strictly impacted due to the phage infection and not cellular growth, which was also occurring during the phage infection. To identify proteins that changed in expression due to cellular growth alone, we compared all permutations of mock-infected time points and identified 147 proteins that were differentially regulated due to cell growth. We called this group Growth (Fig. 3B). Comparing the Growth proteins to the 309 proteins differentially regulated at 75 min postinfection revealed a set of 58 proteins differentially regulated due to both Growth and φX174 infection (Fig. 3B). Removing these 58 proteins along with the 12 proteins shared with the 60-min-postinfection sample gave a set of 239 proteins differentially regulated due solely to phage infection. We called this group Phage (Fig. 3B).

Examining the biological functions enriched within the Growth protein set revealed proteins involved in metabolic activity such as energy production and conversion (C) and amino acid (E) and carbohydrate (G) transport and metabolism (Fig. 3C). In contrast, Phage proteins were enriched for a distinct set of categories: cell wall/membrane/envelope biogenesis (M); intracellular trafficking, secretion, and vesicular transport (U); and defense mechanisms (V) (Fig. 3C).

### Disruption to peptidoglycan synthesis and upregulation of membrane-tethering lipoproteins associated with φX174 protein E expression.

**FIG 4:**
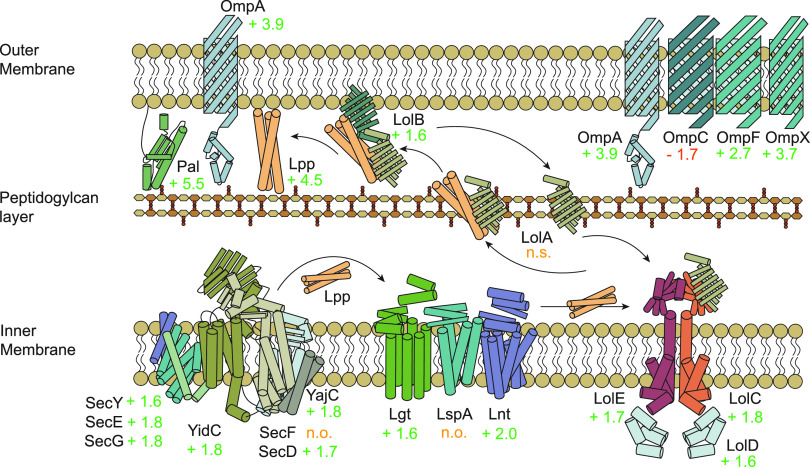
E. coli C122 upregulates membrane-stabilizing and -anchoring proteins Lpp, OmpA, and Pal during φX174 infection. Upregulation of the membrane lipoprotein maturation and transportation pathway. Depicted are nascent lipoproteins translocating across the inner membrane through the Sec pathway, followed by processing, and Lol pathway-mediated export to the outer membrane. Outer membrane proteins are depicted, including OmpA, which has its peptidoglycan-anchoring role highlighted along with Lpp and Pal. Values indicate protein fold change during infection (75P/75C protein samples), n.o, protein not observed; n.s, protein observed but not significantly altered in abundance between conditions.

Looking deeper into the COG category M (cell wall/membrane/envelope biogenesis), we found proteins from the outer membrane lipoprotein maturation and transportation pathway were significantly upregulated. Typically, a protein is translocated across the inner membrane from the cytoplasm by the Sec pathway or via the twin-arginine translocation (TAT) pathway. We did not observe differential regulation of the TAT pathway but did see the upregulation of all components of the cytoplasmic membrane complex of the Sec pathway (SecYEG, FCs +1.6, +1.8, and +1.8, respectively) and the accessory protein YidC (FC +1.8) (27) (Fig. 4). PANTHER overrepresentation analysis of proteins identified as upregulated from the Phage protein set (Data Set S1) highlighted that the cell envelope Sec protein transport complex is significantly overrepresented (*P* value < 0.0002) during infection. This complex facilitates the translocation of nascent unfolded proteins into the periplasm in an ATP-dependent fashion (28). We did not identify differential expression of SecA, an ATPase that interacts with SecYEG and SecB (29), nor the significant differential expression of SecB itself. SecF was not observed, but its binding partner, SecD, was significantly upregulated (FC +1.7). YajC also interacts with SecDF and we saw it significantly upregulated (FC +1.8) to a similar magnitude during φX174 infection (Fig. 4).

After nascent lipoproteins are translocated through the inner membrane via the Sec pathway machinery, a diacylglycerol group is added to the protein by the inner membrane diacylglycerol transferase protein Lgt (30). We observed the significant upregulation of Lgt in the phage-infected samples (FC +1.6) as well as the Lnt protein that subsequently performs N-terminal acylation (FC +2.0) (30), but we did not observe signal peptide cleaving protein LspA (Fig. 4). The ATP-driven LolCDE inner membrane complex (FCs +1.8, +1.6, and +1.7, respectively) releases lipoproteins to the periplasmic chaperone protein LolA (expression not significantly changed) (31). From there, LolA facilitates the lipoprotein’s transfer to the outer membrane receptor LolB (FC +1.6), which mediates their integration into the outer membrane (32) (Fig. 4).

The upregulation of the lipoprotein processing and trafficking machinery was in concordance with the upregulation of the major outer membrane lipoprotein (Lpp) in the Phage protein set (FC +4.5) (Fig. 4). Lpp tethers the outer membrane to the peptidoglycan layer through a covalent linkage, facilitating structural integrity and maintenance of cell shape (33). Lpp attachment to the outer membrane is dependent on LolB (FC +1.6), which, if depleted, is detrimental to the cell due to Lpp mislocalization (34). Similar to Lpp, we saw upregulation of OmpA and Pal (FCs +3.9 and +5.5, respectively), which also function in maintenance of cell membrane integrity and act through noncovalent interactions connecting the outer membrane and peptidoglycan layers (35, 36) (Fig. 4). This result suggests the cell, during φX174 infection, was experiencing envelope integrity problems, possibly of the peptidoglycan layer, resulting in the upregulation of these membrane-anchoring proteins.

Previous work has demonstrated that phage lysis protein E acts to directly disrupt the activity of integral inner membrane protein MraY (37–39). MraY catalyzes the first membrane-associated step of peptidoglycan biosynthesis (40), and interruption of MraY function by protein E is believed to result in the loss of envelope integrity through synthesis inhibition (38). While the complete mechanism is yet to be resolved, lysis protein E is expressed at increasing amounts throughout the infection cycle and the lytic response is coupled to the cell growth rate and nutritional state (41). Furthermore, protein E expression seems to be associated with failed septation (42, 43).

**FIG 5:**
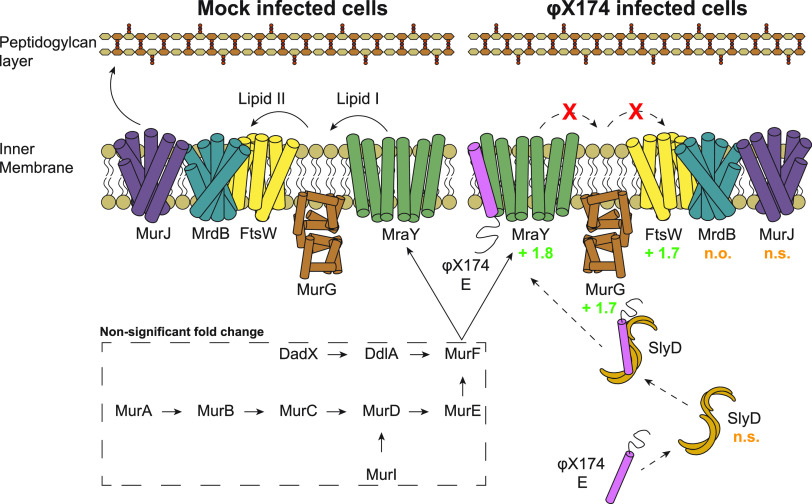
Selective upregulation of the membrane-bound steps of peptidoglycan synthesis observed during infection. Membrane-bound and cytoplasmic proteins involved in peptidoglycan synthesis shown along with φX174 protein E, which inhibits MraY. Values indicate protein fold change during infection (75P/75C protein samples); n.o., protein not observed; n.s., protein observed but not significantly altered in abundance between conditions.

We first observed protein E at 60 min, and by 75 min, expression had more than doubled (Fig. 1C). At 75 min we saw upregulation of MraY (FC +1.8) and all observed downstream inner membrane proteins in this pathway, MurG (FC +1.7) and FtsW (FC +1.7), although we saw no upregulation of the cytoplasmic components of the peptidoglycan biosynthetic pathway that lie immediately upstream of the MraY protein (Fig. 5).

In contrast to the extensive proteome-level response, only 16 differentially expressed RNA transcripts were attributed to COG category M. Within this grouping, only two genes, *murI* and *yjdB*, are involved in lipopolysaccharide biosynthesis. *murI*, which is responsible for lipopolysaccharide core region biosynthesis by catalyzing the racemization of l-glutamate to d-glutamate (44), was downregulated (FC −1.5), while *eptA*, which catalyzes the addition of phosphoethanolamine to the lipid A glucosamine disaccharide (45) was upregulated (FC +1.6).

### Transcriptomics identifies significant upregulation of the PSP stress response operon, revealing additional cellular response to membrane damage.

A major transcriptional response of E. coli C122 to φX174 infection was the upregulation of gene products responsible for maintaining envelope integrity and the proton motive force. This was primarily seen through the upregulation of multiple components of the phage shock protein (PSP) response whereby *pspB* (FC +1.7), *pspC* (FC +1.7), and *pspD* (FC +1.8) in the *pspABCDE* operon, and *pspG* (FC +1.8), were upregulated. Collectively, these genes encode protein products responsible for the binding of PspA to damaged membranes. At the proteome level, significant upregulation was observed only for PspB (FC +2.1), while PspE was downregulated, and no change was observed for PspA, PspC, and PspF. Two other genes, *ycjF* and *ycjX*, associated with the PSP response in some species (46), were also upregulated at both the transcriptional and protein levels (RNA FCs +2.4 and +3.0 [Fig. S2B]; protein FCs +2.6 and +1.7, respectively).

One of the most upregulated genes during infection was *fxsA* (FC +4.4), which was not identified in the proteomic analysis. Its only ascribed function is to competitively sequester PifA in the cytoplasmic membrane, preventing PifA from performing its host suicide mechanism, known as F exclusion. F exclusion disrupts the bacteriophage T7 life cycle by arresting all viral and cellular processes upon PifA interaction with T7 products gp1.2 and gp10A (47, 48). No annotated version of *pifA* is in the available C122 genomes, and it is normally found on the F plasmid, which was not present in the cells in this work.

### φX174 infection significantly alters E. coli metabolic gene expression.

**FIG 6:**
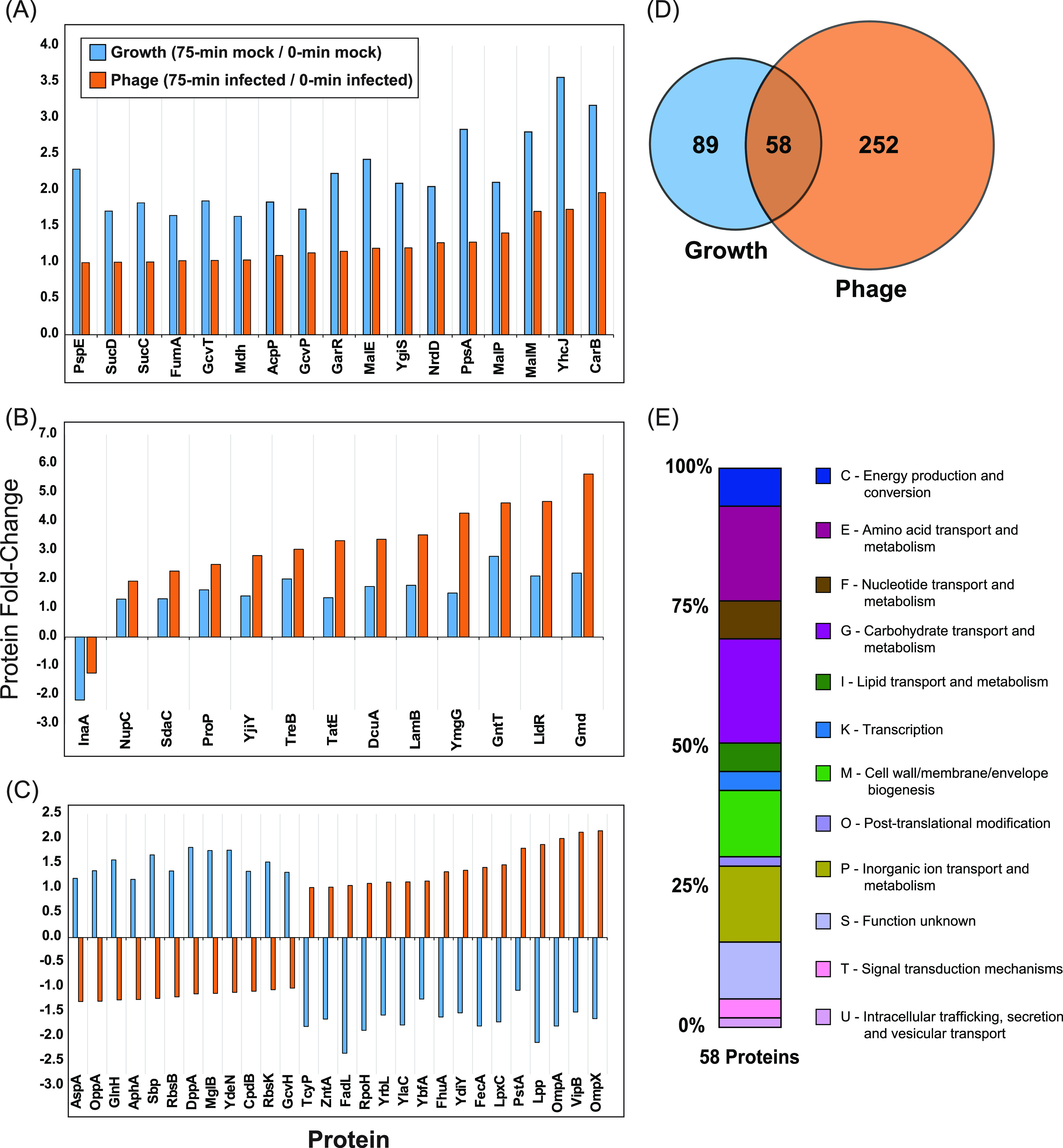
Differential expression patterns of the 58 E. coli C122 proteins shared by Growth and Phage sets. (A to C) Subset of the 58 host proteins where φX174 infection reduces (A), increases (B), or inverts (C) expression. (D) Euler plot of all significantly differentially expressed proteins within the Growth and Phage sets. (E) Clusters of orthologous groups (COG) functional categories used to represent major biological functions and their percent (%) distribution across different groupings for the 58 shared proteins. COGs were assigned to C122 genes using the eggNOG-Mapper (24).

To understand the effects of the phage infection on the 58 proteins detected in both Growth and Phage sets (Fig. 6D), we classified them based on their three distinct patterns of differential regulation (Fig. 6A to C). The first set showed φX174 infection dampening protein expression across 17 genes (Fig. 6A). PANTHER GO biological process analysis of this list found enrichment in proteins involved in the tricarboxylic acid cycle (4 proteins, *P* value = 5.79E−06), indicative of a disruption to overall metabolic activity during phage infection. The second group of genes showed increased expression in the Phage protein set (Fig. 6B). PANTHER GO molecular function analysis on these 14 proteins found enrichment for transporter activity (9 proteins, *P* value = 3.59E−06) or, more specifically, carboxylic acid transmembrane transporter activity (5 proteins, *P* value = 8.50E−06). This is reflective of COG distributions of the entire 58 proteins (Fig. 6E), where we see the majority of the highlighted categories being trafficking or transporter groups. The third case showed inverse protein expression pattern between Growth and Phage sets (Fig. 6C), which is especially intriguing as it would indicate these proteins are particularly important to phage infection. A PANTHER GO biological process or molecular function analysis of these 28 genes revealed no significant enrichment. However, 15 of the 28 proteins are located in the cell envelope (*P* value = 2.82E−11), indicating significant and likely diverse transporter functions from this group of proteins. Of particular interest are aforementioned proteins Lpp and OmpA, as well as OmpA structural homolog OmpX (FC +3.7) (Fig. 4) (49), all of which were found to have large fold change inductions during phage infection but were downregulated during mock infection (Fig. 6C).

Interestingly, OmpA was recently identified as a competitive binder of the lipoprotein RcsF (49), which we also identified as significantly upregulated during phage infection (FC +1.6). RcsF is a positive regulator of the Rcs stress response (50), of which we identified the significant upregulation of Rcs components RcsD (FC +1.7) during infection.

Biological process GO term enrichments of the transcriptomics data showed broad representation of two different host functions which affected host metabolic activity when mapped to COG categories. The first of these host functions corresponded to the COG categories nucleoside transport and metabolism (F), amino acid transport and metabolism (E), and carbohydrate transport and metabolism (G). The COG categories were represented with the enriched biological functions of plasma membrane glucose import (17.9-fold, 4 genes, *P* value = 4.50E−04), glycine catabolic process (14.3-fold, 4 genes, *P* value = 7.77E−04), nucleoside transmembrane transport (11.2-fold, 5 genes, *P* value = 3.59E−04), and nucleobase-containing small-molecule interconversion (5.5-fold, 7 genes, *P* value = 7.54E−04). The second host function mapped to COG categories corresponded to energy production and conversion (C) with the enriched biological functions ATP synthesis-coupled proton transport (11.2-fold, 5 genes, *P* value = 3.59E−04), gluconeogenesis (7.7-fold, 6 genes, *P* value = 4.24E−04), and glycolytic process (6.0-fold, 7 genes, *P* value = 4.81E−04) (Fig. S4).

### φX174 infection strongly activates heat shock chaperone response.

**FIG 7:**
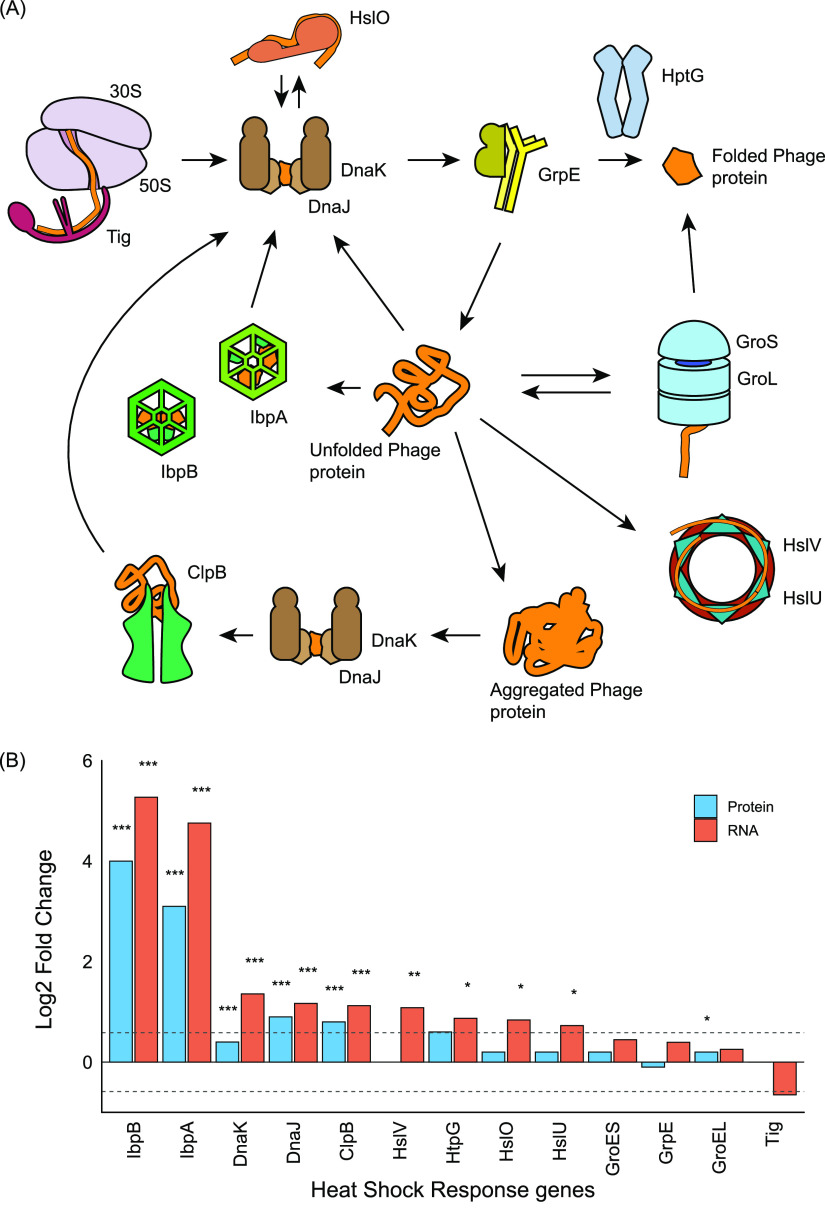
Large changes to gene expression across the heat shock chaperone and protein folding pathway in E. coli C122 during φX174 infection. Proteins depicted in this pathway are transcriptionally regulated by transcription factor RpoH (σ^32^), which we saw upregulated during infection. (A) The chaperone system revealing the process controlling protein folding from synthesis to native protein state. The chaperone trigger factor (Tig) binds to the translating ribosome and the growing peptide chain. The DnaK/J chaperone proteins aid in folding by binding to hydrophobic regions of nascent proteins, preventing their misfolding and aggregation. DnaK/J may bind to HtpG to reactivate inactive protein substrates on the path to native protein fold (93). The holdase HslO binds to unfolded protein, which is released to DnaK for folding (94). GrpE may interact with DnaK, thereby dissociating ADP and releasing the bound protein (95). Small heat shock chaperones IbpA/B bind nascent proteins, preventing their irreversible aggregation into inclusion bodies, and await the availability of folding chaperones DnaK/J and GroEL/ES. The main folding machine of the system is the GroEL/ES complex. This ATP-dependent system facilitates folding of nascent proteins within the complex’s inner surface, releasing proteins in their native state. In the case of protein aggregates produced from improperly or partially folded proteins, the chaperone ClpB mediates their disaggregation for their refolding. Unfolded protein may also shuffle to the HslUV protease/ATPase complex for proteolysis (96). (B) Differential expression of transcripts and proteins was measured at 60 and 75 min, respectively. Dashed lines indicate the log_2_ fold change ± 0.58 values used as significance criteria. *P* values are shown as *** for ≤0.001, ** for ≤0.01, and * for ≤0.05.

The strongest host response we observed at both the protein and RNA level was the heat shock response, led by the small heat shock proteins (sHSPs) IbpA and IbpB (Fig. 1). Our proteomics and transcriptomic data showed that they had the largest increase in expression of any host gene products (Fig. 1C). Both *ibpA* and *ibpB* are controlled by the σ^32^ regulon (51), and their large upregulation during φX174 infection is consistent with the significant increase in RpoH transcription factor σ^32^ (FC +2.0), along with chaperones ClpB (FC +1.7) and DnaJ (FC +1.9) (Fig. 7). Notably, only a subset of the σ^32^ heat shock stress response regulon was activated during φX174 infection, as we observed only 11 proteins and 17 transcripts belonging to this regulon differentially regulated (Fig. 7; Data Set S3). Similarly, the general stress response master regulator, σ^38^ (RpoS), was not differentially regulated, and only a small proportion of proteins (5%) from the genes under its control were differentially regulated during phage infection (Data Set S3).

10.1128/mSystems.00046-21.9 DATA SET S3 σ^32^ and σ^38^ analysis. Download Data Set S3, XLSX file, 0.02 MB. Copyright © 2021 Wright et al. 2021 Wright et al. https://creativecommons.org/licenses/by/4.0/ This content is distributed under the terms of the Creative Commons Attribution 4.0 International license.

Together, these data indicate that φX174 infection was not inducing a general stress response via the σ^38^ or σ^32^ regulon and that there was selective upregulation of members of the heat shock chaperone network (Fig. 7).

## DISCUSSION

In this work, we have comprehensively measured the E. coli C122 host response to φX174 infection at the proteomic and transcriptomic level, representing the first characterization of the dynamics of *Microviridae* infection at this level of detail. We identified 2,184 proteins and quantified all 11 phage proteins and 1,752 host proteins across seven time points spanning the entire infection cycle. Transcriptomics identified significant changes during infection including differential expression of 528 genes, constituting 11% of the total transcriptome, similar to other studies (52).

We found no evidence for substantial host proteome remodeling during the early stages of infection prior to 60 min (Fig. 3A), which aligns with previous work showing that φX174 has a minimal impact on host protein production until just prior to lysis (53). Between 60 min and 75 min, we saw 255 proteins upregulated while only 54 were downregulated, with the most strongly downregulated proteins decreasing faster than would be expected from dilution due to cell growth (see Fig. S5 in the supplemental material). It is well established that phage infection can cause drastic and broad metabolic remodeling of infected cells (11, 54, 55), which is in agreement with what we saw for both our proteomic and transcriptomic data sets.

10.1128/mSystems.00046-21.5 FIG S5 Fold changes of significantly down- and upregulated proteins at 75-min time point across the entire time course. Fifty-four downregulated and 244 upregulated host proteins determined to be significantly differentially regulated (FC ±1.5 and *P* value <0.025) at 75 min post-infection initiation were plotted according to their log_2_ fold changes between infected and uninfected samples across the infection time course. Download FIG S5, PDF file, 2.3 MB. Copyright © 2021 Wright et al. 2021 Wright et al. https://creativecommons.org/licenses/by/4.0/ This content is distributed under the terms of the Creative Commons Attribution 4.0 International license.

We showed that membrane damage responses dominated the host response to φX174 infection (Fig. 4 and 5). We also found membrane lipoprotein maturation and transportation pathway enrichment (Fig. 4), which suggests an upregulation of this pathway in response to the host’s need to traffic certain lipoproteins to the outer membrane, such as major lipoprotein (Lpp). Lpp is one of the most abundant (lipo)proteins of E. coli (56) and is reliant on the Lol pathway for outer membrane insertion (34). We saw Lpp downregulated during growth (FC −2.1) and upregulated during φX174 infection (FC +4.5), thus emphasizing an infection-mediated response. Lpp has recently been shown to regulate cell shape and mechanical rigidity by tethering the outer membrane to the peptidoglycan layer (33), which leads us to suggest that during φX174 infection, Lpp is upregulated to maintain the cell’s envelope integrity.

The most likely model that fits our observations would have lysis protein E from φX174 binding to and inhibiting MraY (37, 38) and causing a decrease in peptidoglycan synthesis which is sensed by the cell and results in upregulation of MraY and inner membrane pathway partners, MurG and FtsW (Fig. 5). The cytoplasmic components of the peptidoglycan pathway and the chaperone protein, SlyD, which stabilizes the φX174 lysis protein E and is thought to promote its interaction with the inner membrane (57), are not upregulated (Fig. 5).

Secondarily, as the φX174-infected E. coli cell continues to grow but is unable to synthesize enough peptidoglycan, the stability of the envelope becomes compromised. The cell senses this and attempts to compensate by upregulating the Lpp, OmpA, and Pal proteins (Fig. 4). OmpA also seems to have a role in maintaining envelope integrity by linking the outer membrane to the peptidoglycan layer through the noncovalent linkage of its C-terminal domain to peptidoglycan (36). In addition to this activity, OmpA has a direct interaction with peptidoglycan stress sensory protein RcsF (49), which we also saw upregulated during infection (FC +1.6). Pal protein is also a mediator of outer membrane and peptidoglycan stability by forming a structural linkage between both layers (35).

Host membrane responses to phage infection are common and can involve responses such as induction of polysaccharide capsule biosynthesis (58) and phage shock response (55). We saw the significant upregulation of components *pspB*, *pspC*, and *pspG* and the potentially related genes *ycjX* and *ycjF* (Fig. S2), presumably in response to the loss of envelope structural integrity during the leadup to lysis. A similar induction of the phage shock response was observed with phage PRD1 infection of E. coli (59) and antibiotics that inhibit peptidoglycan synthesis (60).

Both proteomics and transcriptomics showed a massive upregulation of small heat shock proteins IbpA (FC +8.7) and IbpB (FC +15.6) and their transcripts (FC +27.1 and +38.6, respectively) during infection. The IbpA/B proteins are well described, and the sHSP family is conserved across all domains of life (61). They are typically described as “holdase” proteins, as they function as chaperones binding misfolded or unfolded (nascent) proteins to prevent their irreversible aggregation within the cytosol, while they wait for available ATP-mediated folding machinery (62, 63) (Fig. 7). The large mismatch between *ibpA/B* RNA and protein fold changes in our measurements may be due to the RNA thermometer function encoded within their transcripts that ensures they are maximally translated only at elevated temperatures (64).

We speculate that the upregulation of IbpA/B proteins is a conserved nonspecific bacterial response to viral infection based upon their upregulation both during phage infection (59, 65) and during heterologous protein overexpression (66, 67). Therefore, as a result of their currently defined function (62, 63), IbpA/B upregulation in E. coli C122 during φX174 infection is a host response to higher-than-normal levels of protein production (in this case, phage protein production), and in the absence of available protein folding machinery, irreversible protein aggregation or misfolding is prevented through the upregulation of these holdase proteins.

Phage may also benefit from the specific features of the IbpA/B function by having a queue of proteins held in a near-folded state that can be quickly folded into their final forms by HSP70 (DnaK) and HSP100 (ClpB) activity. Proteins bound by IbpA/B are not spontaneously released and can be released only by DnaK and ClpB (64), introducing a buffering system and control point for virion assembly.

The *ibpA/B* genes have never been identified from genetic screens of host factors necessary for capsid morphogenesis, but sHSPs are encoded in the genomes of cyanophage infecting *Synechococcus* and *Prochlorococcus* groups (68, 69). Furthermore, a bacteriophage-encoded J-domain protein (Rki) has been identified in T4-related enterobacteriophage RB43 that interacts with E. coli J-domain-interacting chaperone protein DnaK (HSP70) and stabilizes the σ^32^ heat shock response (70). MS2 phage relies on DnaJ (HSP40) (71), while the HSP60 chaperones GroEL/ES are required for phage λ, PRD1, HK97, and T7 morphogenesis (72–74). GroEL-like proteins have also been found encoded within newly annotated bacteriophage genomes (75). In *groEL/groES* mutants, phage T4 head morphogenesis is disrupted and results in a random aggregation of phage head proteins attached to the inner membranes (76), pointing to the critical disaggregating and/or folding role of the HSP chaperones in phage capsid assembly. Young et al. (1989) found that lysis sensitivity increased in E. coli when plasmid-encoded φX174 lysis protein E and heat shock genes *dnaK*, *dnaJ*, *groEL*, and *grpE* are present (77). Recently, φX174 has been suggested to use heat shock promoters to co-opt host responses to infection to drive its life cycle (12, 78). Therefore, together with our data, we propose that the host heat shock response and in particular IbpA/B are important components for φX174 replication.

## MATERIALS AND METHODS

### φX174 infection.

Methods are composed in accordance with reference 79. Media and buffer components were from Sigma-Aldrich, unless stated otherwise. E. coli C122 (Public Health England NCTC122) was grown overnight at 37°C/250 rpm in phage-LB (80) and then inoculated 1/100 in fresh phage-LB and grown to mid-log phase (∼0.7 OD_600_) before addition of phage (37°C/250 rpm). Wild-type φX174 (14) was used for all infections of E. coli C122 at a multiplicity of infection (MOI) of 5. Phage infection was synchronized by pelleting mid-log cultures (4,000 relative centrifugal force [RCF]/8 min) and resuspending the pellet to 1/10 of the original growth volume with cold HFB buffer (81). Starved cells were incubated at 16°C/30 min to facilitate phage attachment and to prevent DNA ejection (82). After 30 min, infection was initiated with the addition of phage-LB (37°C) to the original volume. Controls (mock infected) had the same volume of phage-LB and 10 % (vol/vol) glycerol added at mid-log phase as the phage-infected samples.

### Protein purification and tandem mass tagging (TMT) labeling.

Biological triplicates of E. coli C122 were grown to mid-log in a 200-ml culture volume (37°C/250 rpm) and then split into two 100-ml volumes: one to be infected with φX174 at an MOI of 5 and the other mock infected with an equal volume of phage-LB with 10% (vol/vol) glycerol (storage solution of φX174). Culture growth was synchronized with 10 ml of ice-cold HFB, and infection was initiated with 90 ml phage-LB (37°C). At 0, 15, 30, 60, and 75 min postinfection, 12 ml was removed from each culture (without replacement) and placed on ice. At the conclusion of the time course, intact cells were pelleted at 3,220 RCF/8 min. The samples were then suspended in cold 1× phosphate-buffered saline (PBS) and washed an additional two times, discarding the supernatant. Cell pellets were stored overnight at −20°C. Cell pellets were suspended in 500 μl lysis buffer (100 mM Tris-HCl, 1% [vol/vol] sodium dodecyl sulfate, 8 M urea, 1× protease inhibitor cocktail solution [Roche, Switzerland]). Reconstituted cells were lysed with a Branson Sonifier 450 ultrasonic probe with 15 pulses for 15 s at a 30% duty cycle and 30% amplitude. Samples were then pelleted at 6,000 RCF/10 min, and supernatant was moved to a new tube. Proteins were reduced at 37°C/1 h in 10 mM dithiothreitol, followed by alkylation with 30 mM iodoacetamide for 1 h in the dark. Residual iodoacetamide was quenched with an equal molarity of dithiothreitol.

Proteins were precipitated by addition of 2 ml methanol, 0.5 ml of chloroform, and 2 ml of water (all ice cold). Samples were pelleted at 5,000 RCF/10 min, and protein pellet was removed, washed with additional methanol, and dried. Protein pellets were solubilized with 100 mM Tris-HCl/8 M urea. Solutions were pelleted at 12,000 RCF/10 min, and supernatant was collected in a new tube. Samples were diluted to a urea concentration of 1.6 M through addition of 100 mM Tris-HCl, followed by protein quantitation (Pierce bicinchoninic acid [BCA] protein assay kit; ThermoFisher Scientific). One hundred fifty micrograms of protein from each sample was digested overnight/37°C with 1.5 μg trypsin (Promega), followed by addition of a further 1.5 μg of trypsin and digestion for an additional 4 h. Peptides were acidified with formic acid at 1% (vol/vol) and then C_18_ stage-tip purified (83). Peptide samples were dried down using vacuum centrifugation, followed by resuspension in 200 mM HEPES buffer (pH 8.8, adjusted with NaOH). Peptides were quantified (Micro BCA protein assay kit; ThermoFisher Scientific), and 35 μg peptide from each sample was brought up to 140 μl using HEPES buffer. Normalization controls were produced through pooling mock-infected samples (3 μg of peptide per mock-infected sample), designated N.C., and infected samples (3 μg of peptide per infected sample), designated N.P.

Chemical labeling of peptide samples was performed using the TMT 10-plex isobaric label reagent set (ThermoFisher Scientific, USA). An 0.2-mg amount of TMT tag was used to label each sample (see Table S1 in the supplemental material). Note that 128C tag was reserved for N.C. An 11.25-μg amount of N.C. was labeled per channel. Similarly, 131 tag was reserved for N.P. A 5.375-μg amount of N.P. was labeled per channel. After labeling, 8 μl of 5% (vol/vol) hydroxylamine was added to each sample to quench residual tags. Lastly, samples were combined across channels, dried down using vacuum centrifugation, and then purified as described previously (83).

10.1128/mSystems.00046-21.6 TABLE S1 TMT labeling scheme. There were four TMT 10-plex experiments with each channel comprising a different sample, except for the 128C and 131 channels. Channel 128C contained the pooled mock-infected samples (designated N.C.), and channel 131 contained the pooled φX174-infected samples (designated N.P.). Download Table S1, DOCX file, 0.02 MB. Copyright © 2021 Wright et al. 2021 Wright et al. https://creativecommons.org/licenses/by/4.0/ This content is distributed under the terms of the Creative Commons Attribution 4.0 International license.

### High-pH peptide prefractionation.

Dried peptide samples (TMT channels 1 to 4) were suspended in high-pH buffer (5 mM NH_4_OH, pH 10.5), loaded onto an Agilent Zorbax Extend-C_18_ column (3.5-μm bead size, 300-Å pore size, 2.1 mm by 150 mm), and washed with buffer A (0.1% [vol/vol] formic acid, 2% [vol/vol] acetonitrile) for 10 min, followed by elution with an increasing gradient of buffer B (5 mM NH_4_OH, 90% [vol/vol] acetonitrile). The gradient of buffer B was 3% to 30% for 55 min, then to 70% for 10 min, and finally to 90% for 5 min at a flow rate of 300 μl/minute. Samples were collected every minute and pooled into 13 fractions, vacuum centrifuged, and then suspended in buffer A to a concentration of 0.1 μg/μl.

### Mass spectrometry.

Samples were analyzed on a Q-Exactive mass spectrometer (ThermoFisher Scientific) coupled to an Easy-nLC1000 system (ThermoFisher Scientific, USA). Peptide samples were injected onto the liquid chromatography (LC) system using buffer A and were bound on a 75-μm by 100-mm C_18_ Halo column (2.7-μm bead size, 160-Å pore size). A flow rate of 300 nl/minute using an increasing linear gradient of buffer B (0.1% [vol/vol] formic acid, 99.9% [vol/vol] acetonitrile) was run from 1% to 30% for 110 min followed by 85% buffer B for 10 min. The mass spectrometer was operated in top-10 mode, with a full scan set at a resolution of 70,000 (at 400 *m/z*) across the *m/z* range of 350 to 1,850 (isolation window of 0.7 *m/z*), and an automatic gain control (AGC) target of 1e6 (or maximum fill time of 250 ms). Selected precursor ions were transferred from the C-trap to the higher energy collision dissociation (HCD) cell for fragmentation at a normalized collision energy of 35% with precursor dynamic exclusion of 90 s. Tandem mass spectrometry (MS/MS) spectra were collected at a resolution of 70,000 (at 200 *m/z*) with an AGC of 1e5, a maximum injection time of 250 ms, and a fixed first mass of 115.0 *m/z*.

Raw files were exported to Proteome Discoverer v2.1 (ThermoFisher Scientific) for processing. Precursors selected for fragmentation that had greater than 30% interference were excluded from analysis. Methionine oxidation, N-terminal carbamylation, asparagine and glutamine deamidation, N-terminal acetylation, N-terminal glutamic acid to N-pyroglutamine, N-terminal glutamine to N-pyroglutamine, and TMT 10-plex labeling of primary amines were selected as dynamic modifications. Cysteine carbamidomethylation was designated a fixed modification. Minimum peptide length was set at 5. Spectra were searched against a custom E. coli C122 database that included the phage proteins, totaling 3,806 entries (Data Set S4). False-discovery rates were fixed at 1% at the peptide and protein level. Runs were normalized by dividing by all channels (e.g., 126, 128N) by the pooled control (128C), log_2_ transformed, and further normalized by subtracting by the median of each sample group (Table S1). Sample infected 60_1_ was excluded from statistical tests due to phage proteins being designated outliers by OutlierD quantile regression analysis (84). Host proteins lacking enough observations for quantitation at any time point or condition were not processed further. Student’s *t* test was performed on the log_2_-transformed data. Due to effects of ratio compression and to appropriately control for false discovery in a (low-power) medium-scale experiment (85), significance was deemed with a protein fold change cutoff ≥±1.5, *P* value <0.025.

### RNA isolation and RNA-seq.

Biological triplicates of E. coli C122 were grown and infected with wild-type φX174 at MOI = 5. Phage infection was synchronized as for proteomics sampling. Following cell resuspension in 37°C LB medium, 5-ml samples were removed from infected and mock replicates at initial infection (time = 0 min) and at 60 minutes after infection initiation. Samples were pelleted (3,500 RCF/5 min/4°C) and resuspended in 200 μl 1× PBS, 400 μl of RNAprotect bacterial reagent (Qiagen: 76506) was added, and the mixture was vortexed and incubated for 10 min at room temperature. Following protection, the RNeasy minikit (Qiagen; 74106) was used with the optional DNase step. RNA samples were then eluted, and RNA was quantified (Qubit RNA HS assay kit; ThermoFisher Scientific; Q32852) and stored at −80°C. Sequencing library preparation was performed by Macrogen Inc. (South Korea). The rRNA from samples was depleted with a Ribo-Zero kit (Illumina), and the RNA library was generated with a TruSeq stranded mRNA kit for microbes. Libraries were sequenced with Illumina HiSeq 2500 (2 × 100-bp mode). The PhiX control library was not used during the sequencing run to avoid cross-contamination.

Read quality was checked with FastQC followed by Rsubread analysis using the standard protocol (86). BAM files were processed with the Rsubread featureCount function against a manually generated General Feature Format (GFF2, or GTF) file derived from the NZ_LT906474.1 RefSeq GFF3 file. RNA differential expression was analyzed using DESeq2 (87) and visualized using the ggplot2 R package (88). To analyze trends in differential expression, gene names were converted to UniProt identifiers using the Retrieve/ID mapping tool (89) and searched against the PANTHER database gene list analysis tool (90) using the statistical overrepresentation test against the internal reference Escherichia coli gene list to generate overrepresented GO terms for both cellular compartments and biological processes. The converted UniProt IDs were used to extract individual GO terms as before for all genes in the differentially expressed bins using the EcoCyc database (91).

### Databases and gene analysis.

E. coli C122 gene annotations for proteomics experiment and COG assignments were produced through the eggNOG-Mapper using default parameters (24). For σ^32^ and σ^38^ transcription factor assignments, we extracted orthologous gene members from the gold-standard E. coli K-12 gene annotations from the EcoCyc database (91) and assigned matching annotations to C122 genes (Data Set S3). The PANTHER (25) database’s statistical overrepresentation tests and functional classifications were used to map protein and transcript enrichments.

### Data availability.

Mass spectrometry proteomics data have been deposited to the ProteomeXchange Consortium via the PRIDE (92) partner repository with the data set identifier PXD021681.

10.1128/mSystems.00046-21.10 DATA SET S4 MS protein database. Download Data Set S4, TXT file, 1.9 MB. Copyright © 2021 Wright et al. 2021 Wright et al. https://creativecommons.org/licenses/by/4.0/ This content is distributed under the terms of the Creative Commons Attribution 4.0 International license.
