# Supplementary figures and images for: Proteomic and Transcriptomic Analysis of Microviridae φX174 Infection Reveals Broad Upregulation of Host Escherichia coli Membrane Damage and Heat Shock Responses
Source: mSystems. 2021 May 11;6(3):e00046-21. doi: 10.1128/mSystems.00046-21 (PMC8125068; doi:10.1128/mSystems.00046-21)

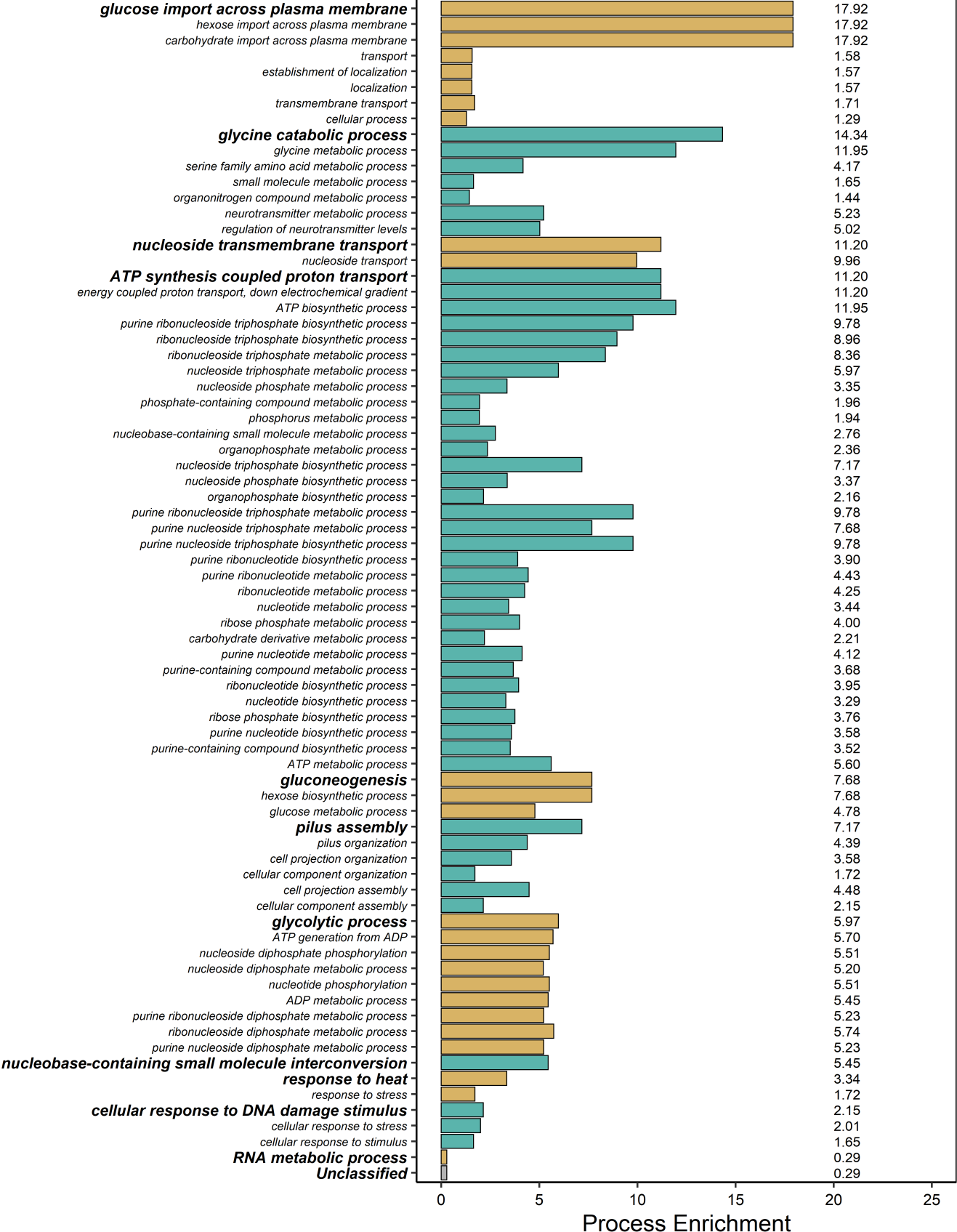

Supplement: FIG S4 [file mSystems.00046-21-sf004.pdf]

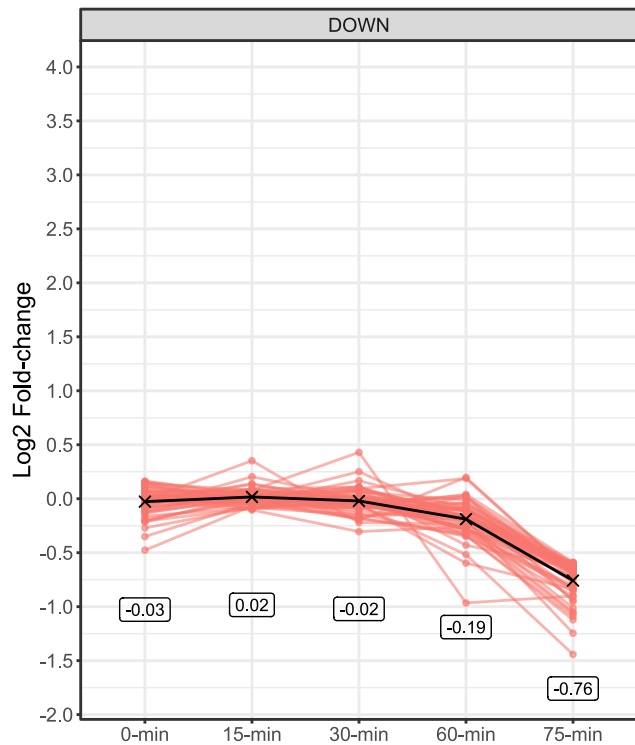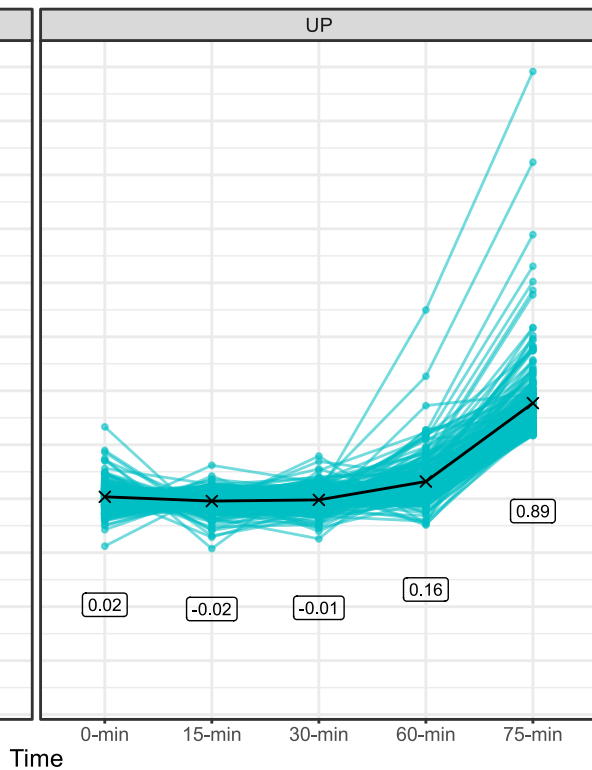

Supplement: FIG S5 [file mSystems.00046-21-sf005.pdf]
