# Supplementary material for: Proteomic and Transcriptomic Analysis of Microviridae φX174 Infection Reveals Broad Upregulation of Host Escherichia coli Membrane Damage and Heat Shock Responses
Source: mSystems. 2021 May 11;6(3):e00046-21. doi: 10.1128/mSystems.00046-21 (PMC8125068; doi:10.1128/mSystems.00046-21)
Supplement: TABLE S1 [file mSystems.00046-21-st001.docx]

|  | Mock-infected | | | | | φX174-infected | | | | |
| --- | --- | --- | --- | --- | --- | --- | --- | --- | --- | --- |
| **TMT** | **126** | **127N** | **127C** | **128N** | **128C** | **129N** | **129C** | **130N** | **130C** | **131** |
| **1** | 0_1_ | 15_1_ | 30_1_ | 60_1_ | N.C | 0_1_ | 15_1_ | 30_1_ | 60_1_ | N.P |
| **2** | 75_1_ | 0_2_ | 15_2_ | 30_2_ | N.C | 75_1_ | 0_2_ | 15_2_ | 30_2_ | N.P |
| **3** | 60_2_ | 75_2_ | 0_3_ | 15_3_ | N.C | 60_2_ | 75_2_ | 0_3_ | 15_3_ | N.P |
| **4** | 30_3_ | 60_3_ | 75_3_ |  | N.C | 30_3_ | 60_3_ | 75_3_ |  | N.P |

Subscript = replicate number
